# The psychosocial impact of alopecia in men: A mixed‐methods survey study

**DOI:** 10.1002/ski2.420

**Published:** 2024-06-29

**Authors:** Fabio Zucchelli, Abbi Mathews, Nick Sharratt, Kerry Montgomery, Jen Chambers

**Affiliations:** ^1^ The Centre for Appearance Research University of the West of England Bristol UK; ^2^ Formerly at the Centre for Appearance Research University of the West of England Bristol UK; ^3^ Formerly at Alopecia UK (Registered charity no. 1111304) Shipley UK

## Abstract

**Background:**

The most common forms of hair loss in men, alopecia areata (AA) (an autoimmune condition) and androgenetic alopecia (AGA) (pattern baldness), alter individuals' appearance in ways that may impact psychological and social wellbeing. We currently have a limited understanding about this impact of alopecia in men, their support needs, and preferences.

**Objectives:**

We sought to investigate and explore the psychosocial impact of alopecia on men, alongside their experiences of treatment and support.

**Methods:**

The study used a mixed methods cross‐sectional online survey with 177 men aged 17–79: 83 with AGA and 94 with AA. Quantitative questions included purpose‐made rating scales of men's support experiences, and standardised measures of wellbeing and appearance‐focused anxiety. Qualitative data comprised participants' answers to an open‐ended question asking about their subjectively salient experiences related to their alopecia.

**Results:**

The combined findings indicate that while participants in both subsamples had sought minimal support for psychosocial concerns, such concerns were in fact commonplace. Over half of participants (56%–57%) shared qualitative accounts of depleted confidence, while wellbeing scores were on average lower than matched norms. Participants identifying as sexual minority also reported greater appearance‐focused anxiety compared to those identifying as straight.

**Conclusions:**

The apparent contrast between participants' minimal help‐seeking and accounts of affected wellbeing suggests an unmet support need for men with alopecia. Masculine norms may impede men from accessing psychosocial support, both by discouraging help‐seeking behaviours and by encouraging minimisation of appearance concerns. The findings also suggest sexual minority status may pose a greater risk of distress in affected men.

## Graphical abstract

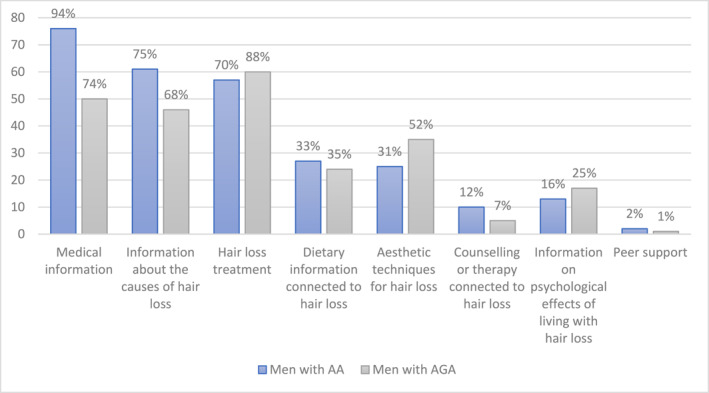

**What is already known about this topic?**

- Hair is an important component of masculinity and body appreciation in men, suggesting hair loss in its two most common forms (androgenetic alopecia [AGA] and alopecia areata [AA]) may have detrimental psychosocial impacts on affected men.

**What does this study add?**

- The findings of this mixed methods (qualitative and quantitative) online survey of men with AGA and AA suggest that, although men from both groups did report depleted confidence and lower‐than‐norm‐matched wellbeing, very few had sought support for psychosocial concerns.

## 1 INTRODUCTION

Alopecia refers to any form of hair loss. The two most common types of alopecia are androgenetic alopecia (AGA) and alopecia areata (AA). AGA, which typically involves gradual loss or thinning of hair in a horseshoe shape on the scalp, is very common, with up to half of men affected by age 50.^1^ AA is an autoimmune condition with an unclear aetiology, thought to contain a genetic component, and has a lifetime prevalence of around 2.6%.^2^ In AA, hair loss usually presents as patches on the scalp, but can progress to total scalp hair loss (alopecia totalis) or the whole body including eyebrows and eyelashes (alopecia universalis). Although up to 80% of individuals with AA experience remission, hair loss and regrowth is often unpredictable,^3^ while men with AGA rarely experience spontaneous regrowth.^1^ Evidence‐based interventions for AGA include oral medications and hair transplantation^4^; corticosteroids and phototherapy are most commonly used for AA,^5^ with limited efficacy. Cosmetic strategies including wigs and medical tattooing are popular in women,^6^, ^7^ but research into men's use of these techniques is limited.

Despite key differences between hair loss patterns and treatments for AA and AGA, research into their psychosocial effect in mixed gender samples suggests commonalities. Those with AA and AGA report psychological impacts, namely higher appearance dissatisfaction, lower self‐esteem, anxiety and depression, which can lead to impaired relationships and isolation.^8^, ^9^, ^10^ Individuals with AA can also experience enacted stigma from others who may assume they are unwell, with more severe hair loss resulting in greater stigma.^11^ While there is limited research comparing psychosocial outcomes in AA and AGA, one study found mixed results between the groups in hair‐related quality of life factors, and no differences in stigmatisation or quality of life.^12^

AA displays similar prevalence among women and men,^2^ and AGA is more common in men.^1^ Despite this, much of the current research into the psychosocial impact of alopecia, and especially AA, focuses on women. Research with mixed gender samples often underrepresents men^13^ and describe findings with limited relevance to men, such as wig use and loss of femininity, despite findings recognising the emotional and psychosocial impact on men.^8^, ^14^ We therefore currently have minimal understanding of the needs and preferences regarding treatment and support in men with alopecia.

Although research suggests women with alopecia may be at greater risk of adverse psychosocial impacts than men,^15^ we cannot assume men are unaffected by appearance pressures, especially as men may often minimise their appearance concerns.^16^ Compared to women, men are on average more reluctant to show concern about health, and engage less with healthcare professionals.^17^ Both issues ‐ minimisation of appearance concerns and limited help‐seeking in men—have been attributed at least in part to masculinity norms that valorise stoicism and self‐reliance.^16^, ^17^ Indeed, with hair identified as an important component of masculinity and body appreciation, alopecia may represent a particularly difficult appearance‐affecting outcome for men in terms of psychosocial adjustment.^18^

It is also unknown how other individual factors, such as sexuality, may influence the impact of alopecia on men. With previous research showing that men identifying as gay or bisexual experience greater appearance pressures compared to those who identify as heterosexual,^18^ sexual minority status could potentially compound the psychosocial impact from alopecia on men.

In the current study, we aim to better understand the support needs and preferences of men with alopecia. To do so, we use a mixed‐methods online survey with the purpose of triangulating quantitative and qualitative findings. We quantified men's support needs and preferences directly by asking which sources of information and support they had accessed, how helpful these were, and what type of information and support they had sought. We indirectly evaluated their support needs by measuring the psychosocial impact of alopecia on men in terms of global wellbeing and appearance‐focused anxiety. To better understand reasons behind men's needs and preferences, we gathered men's qualitative, subjective accounts of hair loss.

## 2 MATERIALS AND METHODS

### 2.1 Design

This study was part of a larger research collaboration between academic researchers and the charity Alopecia UK. We conducted a mixed‐methods online survey, with input from four male public contributors with alopecia on the design, content and delivery of the survey, in order to optimise the survey's appeal and suitability to the study population.^19^ An example of content to which the men contributed was the list of support and information sources to present to participants (e.g., including vlogs and hairdressers as sources). Three contributors had AA and were approached via Alopecia UK, and one had AGA, who was known professionally to the authors.

### 2.2 Procedure

Recruitment was carried out by researchers and Alopecia UK during October 2020–February 2021 mid‐COVID‐19 pandemic. This used snowball and convenience sampling, by sharing tailored versions of the same study advertisement on their website, support groups, and social media.

Participants were presented with an information sheet and consent checklist before accessing the main survey questions, which covered participants' demographics; alopecia background (type and duration of alopecia, and hair loss changes in the preceding 12 months); support experiences and preferences (by selecting all the sources of information and support that they had accessed from a list, selecting from the list of reasons why they accessed them, and rating the helpfulness of the sources on a 5‐point scale from *very helpful* to *very unhelpful*); and standardised psychosocial measures (detailed below). Participants were then asked to share any personally meaningful experiences stemming from their alopecia via an open‐ended question. In this question, participants were invited to share “anything that's important to you about your experiences of hair loss” with the prompts “effects of hair loss on: social life; work/education; leisure time; romantic relationships; general wellbeing; how you feel about yourself.”

The standardised measures used were the Fear of Negative Appearance Evaluation Scale ([FNAES; Ref. 20]; and the Office for National Statistics (ONS) 4 Measures of Personal Wellbeing [ONS4; Ref. 21]).

The FNAES contains six items rated on a 5‐point scale (1 = *not at all*; 5 = *extremely*) in relation to questions about their worries that others will negatively evaluate their appearance, such as “I am concerned about what other people think of my appearance”. Totalled item scores span 6–30, with higher scores suggesting greater appearance‐focused anxiety. The FNAES shows good reliability in people living with an appearance‐altering condition,^22^ and strong internal consistency in this study (*α* = 0.95).

The ONS4 measures life satisfaction, happiness, anxiety and sense of purpose as discrete single‐item measures on a scale of 0–10, (0 = *not at all*; 10 = *completely*). The ONS4 is widely used within the United Kingdom (UK) Annual Population Survey with good predictive and test‐retest reliability and validity, and provides regular population norms by gender and age.^21^

### 2.3 Participants

Participants had to identify as a man, be over 16 years of age, self‐report as having AA or AGA and be based in the UK. There were no further exclusion criteria. In total, 177 men took part: 94 with AA and 83 with AGA. Sixty‐seven per cent of men with AA (*n* = 72) were recruited via Alopecia UK's social media, whereas 55% of men with AGA (*n* = 46) were recruited via Reddit.

Men were predominantly white, heterosexual and educated to degree level. While black and Asian ethnic backgrounds were markedly underrepresented compared to the national population, men identifying as gay or bisexual were overrepresented (16% in our sample vs. 4% nationally; ONS, 2023). The subsample of men with AA was on average 10 years older (44.26; SD = 14.64) than men with AGA (34.14; SD = 12.67), with a broader and more normally distributed age range. A summary of participants' demographic information is in Table 1.

**TABLE 1:** Demographic status of participants.

Demographic category	AA	AGA	Total
Ethnicity/race			
Asian/Asian British	5 (5.3%)	4 (4.8%)	9 (5.1%)
Black/African/Caribbean/Black British	1 (1.1%)	1 (1.2%)	2 (1.1%)
Mixed/multiple ethnic groups	3 (3.2%)	1 (1.2%)	4 (2.3%)
White	87 (92.6%)	75 (90.4%)	162 (91.5%)
Rather not say	0 (0%)	1 (1.2%)	1 (0.6%)
Sexual orientation			
Straight/heterosexual	79 (84.9%)	65 (79.3%)	114 (81.4%)
Gay	9 (9.7%)	9 (11%)	18 (10.2%)
Bisexual	3 (3%)	7 (8.5%)	10 (5.6%)
Other sexual orientation	2 (2.2%)	1 (1.2%)	3 (1.7%)
Relationship status			
Single	24 (25.5%)	39 (47.6%)	63 (35.6%)
Married/civil partnership	35 (37.2%)	12 (14.6%)	47 (26.6%)
Dating or living with partner	22 (23.4%)	26 (31.7%)	48 (27.1%)
Separated or divorced	10 (10.6%)	3 (3.7%)	13 (7.3%)
Widowed	2 (2.1%)	1 (1.2%)	3 (1.7%)
Rather not say	1 (1.1%)	1 (1.2%)	2 (1.1%)
Highest education level			
Postgraduate degree	21 (22.6%)	16 (19.5%)	37 (20.9%)
Undergraduate degree	25 (26.9%)	43 (52.4%)	68 (38.4%)
Vocational qualification (e.g. diploma/certificate)	24 (25.8%)	13 (15.9%)	37 (20.9%)
Secondary school	23 (24.7%)	10 (12.2%)	33 (18.6%)
Occupational status			
Employed full time (including furloughed)	61 (64.9%)	54 (65.9%)	115 (65%)
Employed part time (including furloughed)	9 (9.6%)	3 (3.7%)	12 (6.8%)
Student	3 (3.2%)	16 (19.5%)	19 (10.7%)
Carer	3 (3.2%)	1 (1.2%)	4 (2.3%)
Unemployed	6 (6.4%)	7 (8.5%)	13 (7.3%)
Unable to work	3 (3.2%)	0 (0%)	3 (1.7%)
Retired	8 (8.5%)	0 (0%)	8 (4.5%)
Rather not say	1 (1.1%)	1 (1.1%)	2 (1.1%)
Occupation type (of those employed or retired)			
Senior manager or administrator	9 (11.5%)	5 (8.8%)	14 (7.9%)
Traditional professional (e.g. accountant, solicitor)	12 (15.4%)	13 (22.8%)	25 (14.1%)
Modern professional (e.g. teacher, social worker)	30 (38.5%)	25 (43.9%)	55 (31.1%)
Middle or junior manager (e.g. office manager)	14 (17.9%)	8 (14%)	22 (12.4%)
Technical and craft occupation (e.g. mechanic)	6 (7.7%)	4 (7%)	10 (5.6%)
Clerical and intermediate occupations (e.g. PA)	3 (3.8%)	2 (3.5%)	5 (2.8%)
Semi‐routine manual and service occupations	3 (3.8%)	0 (0%)	3 (1.7%)
Routine manual and service occupations (e.g. cleaner)	1 (1.3%)	0 (0%)	1 (0.6%)

### 2.4 Data analysis

For the categorical questions on support sources and types of support accessed, we recorded frequencies and percentages of participants selecting each item. In the specific helpfulness questions relating to support accessed, we additionally calculated means and standard deviation. For the psychosocial variables of wellbeing and appearance‐focused anxiety, we recorded means and standard deviations for the ONS4 and FNAES. Five multiple imputations, using a predictive mean matching model based on demographic data, were created to model the missing data from 11 men (6%) for the ONS4 and FNAES (whom had entered data for other variables), and available case analysis was used for the categorical variables relating to support sources and type.^23^ Normative ONS4 means from our data collection period were extracted from the ONS. We were unable to perform statistical comparison of ONS4 scores with ONS norms as no standard deviation data were provided for the latter. Owing to the sizeable age divergence between men with AA and AGA in our sample, we did not run any formal statistical analyses comparing the two groups.

Due to the high representation of gay and bisexual men in the sample, we ran an exploratory analysis using independent samples *t* tests (based on unequal variances between groups) to test whether men who identified as straight reported significantly more favourable FNAES scores compared to those identifying as gay or bisexual.

The qualitative data in response to the open‐ended question were analysed by the first and third authors using inductive content analysis, following a published procedure.^24^ This included: The first and third authors co‐creating a codebook (comprised of superordinate categories and subordinate themes characterising patterned responses across participants) after both read all data; establishing an intercoder reliability of >80% in which both authors separately coded the same 20% of the data by mapping participants' extracts to the themes and subthemes; then the first author independently coding the remaining 80% of data. The two researchers shared a critical realist perspective, whereby there is assumed to exist an objective reality beyond human apprehension, which can only be imperfectly signalled through participants' accounts.^24^ For example, in the theme “Breakthrough from shaving head”, the authors acknowledge this as representing the subjective attributions made by participants with AA and AGA, pointing to but not directly representing reality. Both authors kept reflexive logs throughout the research process to raise self‐awareness of how their subjectivities (e.g., assumptions about hair loss in men) may affect their coding, and to mitigate the influence of such subjectivities.

### 2.5 Ethics

Ethical approval was gained from the University of West of England's Faculty Research Ethics Committee (HAS.20.07.201).

## 3 RESULTS

### 3.1 Alopecia background

Of men with AA, just over half (55%; *n* = 52) had alopecia universalis and 10% (*n* = 9) had alopecia totalis, with the remainder (35%; *n* = 33) reporting patchy AA. On average, men with AA had developed hair loss at an older age (mean = 29.89; SD = 15.34) than men with AGA (21.58; SD = 5.81). Men with AA had lost their hair a greater number of years prior to taking the survey (mean = 14.21; SD = 13) than men with AGA (mean = 12.7; SD = 10.7). Over the year preceding survey completion, 31% of men with AA reported experiencing increased hair loss, 55% reported no real changes in hair loss, and 14% had fluctuating hair loss. In men with AGA, 65% reported hair loss, 29% had no real changes, and 6% had fluctuating hair loss over the same period.

### 3.2 Support preferences

**FIGURE 1:**
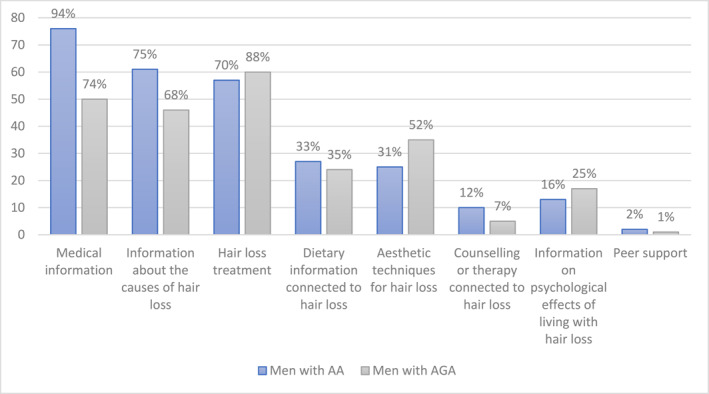
Types of information and support sought by men with alopecia.

Eighty‐six per cent of men with AA (*n* = 81) and 82% of men with AGA (*n* = 68) agreed with statements that they had sought out information, treatment or help concerning their hair loss. Of those who indicated they had not sought help (16%; *n* = 28), the most endorsed reasons across both subsamples were that they did not feel they needed help (6%; *n* = 10) and that they did not think that anyone could help (8%; *n* = 14). Not knowing what help was available was endorsed by four men (2%) with AA. Figure 1 shows the frequencies and percentages (within the subsamples of men with AA and AGA) for types of information and support sought by participants, from presented options.

**FIGURE 2:**
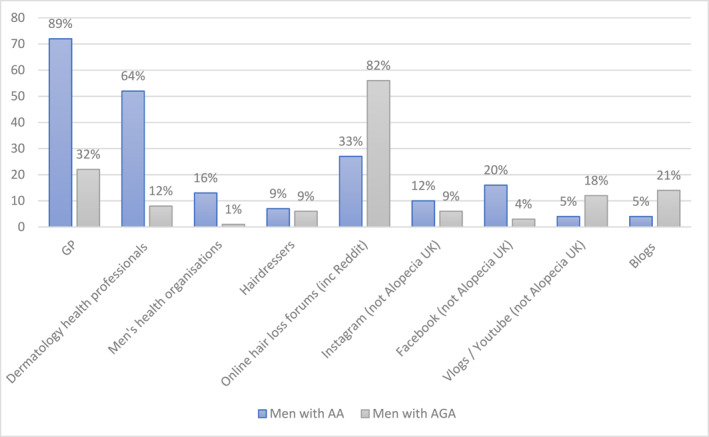
Sources of information and support accessed by men with alopecia.

Figure 2 shows the sources of information and support presented to participants, and the number who reported accessing each source (with only those selected by over 5 from each subsample shown).

Participants were asked to rate how helpful each of the support sources they had accessed had been on a 5‐point scale (1 = *very unhelpful*, 5 = *very helpful*). Table 2 shows the percentages of men with AA and AGA who rated these forms as helpful or unhelpful.

**TABLE 2:** Helpfulness ratings for each support source.

Support source	AA			AGA		
Source of information/support	Mean (SD)	% Rated as helpful^b^	% Rated as unhelpful^b^	Mean (SD)	% Rated as helpful^b^	% Rated as unhelpful^b^
GP	2.67 (1.05)	25%	43%	2.64 (1.26)	27%	55%
Dermatology health professionals	2.82 (1.36)	36%	46%	N/A^a^	N/A^a^	N/A^a^
Online hair forums	3.32 (0.85)	40%	16%	3.38 (1.18)	59%	25%
Men's health organisations; Hairdressers; Instagram; Facebook; Vlogs; Blogs	N/A^a^	N/A^a^	N/A^a^	N/A^a^	N/A^a^	N/A^a^

^a^ Scores are reported only where 20 or more participants responded.

^b^ Helpful ratings include ‘very’ and ‘quite’ helpful; unhelpful ratings include ‘very’ and ‘quite’ unhelpful.

### 3.3 Psychosocial outcomes

From participants' responses to the FNAES, on average, men with AA reported being ‘moderately’ concerned about others negatively evaluating their appearance (mean = 18.34; SD = 7.82). The mean score in men with AGA indicated that on average they reported being between ‘moderately’ and ‘very’ concerned about others' negatively evaluating their appearance (mean = 20.13; SD = 7.81).

Table 3 shows participants' mean scores and corresponding thresholds on the ONS4 for the two subsamples, and for matched national population norms.

**TABLE 3:** Participants' ONS4 scores.

	AA	AGA	National norms^a^
Wellbeing category	Mean (SD) (threshold)	Mean (SD) (threshold)	Mean (SD) (threshold^b^)
Life satisfaction	5.91 (2.99) (medium)	5.70 (2.44) (medium)	7.38 (high)
Purpose	6.40 (3.08) (medium–High)	6.10 (2.56) (medium–High)	7.62 (high)
Happiness	5.79 (2.87) (medium)	5.50 (2.45) (medium)	7.30 (high)
Anxiety	4.28 (2.95) (medium)	4.96 (2.79) (medium)	3.13 (low‐medium)

^a^ National averages for men, time‐matched from Oct 2020‐March 2021, mean age 47.05 (Email communication, Office for National Statistics, received 18 January 2022).

^b^ In life satisfaction, purpose and happiness categories, 0–4 represents a low score, 5–6 a medium score, 7–8 a high score and 9–10 a very high score. On the anxiety item, 0–1 is a very low score, 2–3 is a low score, 4–5 is a medium score and 6–10 is a high score.

### 3.4 Lived experience

The open question on participants' lived experience of alopecia was answered by 136 men (57% of whom had AA). Through the content analysis, the researchers arranged the data into six superordinate categories, with 16 subordinate themes. These are outlined alongside the frequency of participants whose accounts were coded to each theme in Table 4, split into men with AA versus AGA. Full definitions of each theme are provided in Table S1 (in Appendix). The most commonly coded theme was ‘Depleted confidence & wellbeing’, covering 56%–57% of the sample. Other prevalent themes included ‘Coming to terms (with hair loss)’, ‘(using) Concealment strategies’ and ‘Perception of diminished attractiveness’ (14%–28% across each).

**TABLE 4:** Themes and example quotes following content analysis.

Category	Theme	Example quotes	AA *n* (%)	AGA *n* (%)
*Internal experiences*	Depleted confidence & wellbeing	*AA: “Has knocked my confidence. Always concerned people are staring at my alopecia.”; “I despised seeing myself without hair… I was determined not to let those feelings affect my day to day life but it was hard”*	42 (57%)	33 (56%)
		*AGA: “I'm very conscious about my hair and other people hair. For example, if I see another bald guy I immediately start judging his looks and compare it to my look.”*		
	Coming to terms	*AA: “…all in all, having suffered from the condition for over twenty years, at least 15 of which have included total hair loss, I'm pretty comfortable with how I am now. Life's too short.”*	11 (15%)	14 (24%)
		*AGA: “…whilst I'm conscious of [my hair loss] I don't feel it holds me back. It also does get easier as you get older.”*		
	Perception of diminished attractiveness	*AA: “[Hair loss has] completely ended my love life. Having some regrowth but can't go on a date. Generally I'm ok with it apart from my perception of my own attractiveness. It's trashed.”*	10 (14%)	17 (28%)
		*AGA: “I feel people judge me on my hair loss. That I look older and that I look physically less attractive because of it.”*		
	"I don't recognise myself"	*AA: “I still get very shocked/distressed when I look in a mirror.”; “I often forget what I look like and then catch myself in a window and for a split second don't recognise myself.”*	12 (16%)	5 (8%)
		*AGA: “Overall I think losing hair really changes how you see yourself, and your identity for lack of a better term.”*		
	Non‐scalp hair loss especially difficult	*AA: “I was quite a hairy man and to lose every last hair on my body and not being able to grow a beard feels like my masculinity has been stripped away.”*	8 (11%)	0 (0%)
	Personal growth through hair loss	*AA: “I have been bald for many years so have grown to live with how I look and how people react. It is not something I'd wish on anybody but it does make you strong, self reliant and not hung up about trivia. It does affect your confidence initially, but after a while you learn that people who judge on appearance are pretty shallow.”*	5 (7%)	1 (2%)
*Practical experiences*	Social functioning impacted	*AA: “I do not want to do anything that consists of going out without a hat. I will probably not go out on a night out with friends until it grows back.”*	19 (26%)	4 (7%)
		*AGA: “Hair loss has exacerbated every negative feeling I have about myself. I avoid socialising where possible and wear a cap when able.”*		
	Concealment strategies	*AA: “I ended up growing my hair to hide my hair loss… If I had my previous short hair cut, I would be very anxious about it. Growing my hair has helped me forget about it.”*	18 (24%)	8 (14%)
		*AGA: “I cut my hair short during lockdown 1 and it revealed my hairline was majorly receding. Since then I've tried to keep it long to disguise it but I've felt persistently anxious about it.”*		
	Work/education impacted	*AA: “[My hair loss] got progressively worse once I entered 6th form, and left at the end of the first year… I've tried multiple times to go back… but I always get anxious predominantly about my hair, and my appearance…”*	7 (9%)	3 (5%)
		*AGA: “I've lost all my confidence and don't go for certain jobs I would be good at.”*		
	Physical difficulties from hair loss	*AA: “I really find physical exercise in gyms a no go as straight away once I'm worked up and sweating everything is noticeable.”; “Eyelashes and nose hair are surprisingly useful and keep a lot of crap out of your eyes and nose.”*	7 (9%)	0 (0%)
	Breakthrough from shaving head	*AGA: “I felt worse trying to hide my hair loss. Recently shaved it entirely and feel much better. Attempting to cover it caused more problems.”*	2 (3%)	7 (12%)
*Influence of others*	Social support	*AA: “I was fortunate to have a loving long‐term partner (now wife) who supported me well throughout.”*	10 (14%)	5 (8%)
		*AGA: “Going bald… It made me feel very self conscious around my friends. But once I joined a rugby team and began to gain confidence it hasn't affected me as much anymore…”*		
	Unsolicited attention	*AA: “… I would get random strangers come up to me on nights out asking what happened last night? … They said that my mates must have shaved my head in my sleep as a joke.”*	9 (12%)	5 (8%)
		*AGA: “What made [hair loss] harder was that even though there's nothing you can do about the situation, baldness is seen as fair game to be made fun of or commented on by mates, work colleagues or even family.”*		
	Hair loss misunderstood	*AA: “Lots of people have said “its not to bad for a man, worse for a woman”. I can't comment on that but losing your hair and your body hair is difficult to get used too especially when you don't feel medically supported.”*	7 (9%)	4 (7%)
		*AGA: “Family and friends say it shouldn't be a problem for me but no matter what they say it does bother me.”*		
	Assumptions of illness	*AA: “I worry about seeing people I know, but not well, because I think they are going to assume I have lost my hair due to chemotherapy, and will avoid me whereas before we may have chatted.”*	5 (7%)	0 (0%)
*Comparators*	Hair loss hardest when young	*AA: “Beyond doubt wearing a wig during puberty impacted on my well being and help build unhealthy coping mechanisms, these rules and triggers are still around today, albeit with less frequency.”*	9 (12%)	10 (17%)
		*AGA: “My hair loss has far less (almost zero) impact on my life now as a 50 year old man than it did when I was in my 30s, 20s, or teenage years.”*		
	AA & AGA differences	*AA: “Because of male pattern baldness people do not take alopecia in men seriously, yet the speed of hair loss, the patches (non uniformed way it disappears) and loss of facial hair made this far worse for me”*	5 (7%)	0 (0%)
*Treatment*	Unsatisfactory treatment	*AA: “My hair loss started quite suddenly in February this year. GP says most likely explanation is reaction to flu like viruses I had in October 2019 and January 2020. It's frustrating that it cannot be tied down more specifically.”*	7 (9%)	9 (15%)
		*AGA: “I've looked into finasteride but it seems expensive and the potential side effects aren't worth the risk. Hair transplants as well are far too expensive.”*		
	Helpful treatment	*AGA: “I've mostly reversed [my hair loss] with Minoxidil. I wish more people knew about the cost‐effective treatments for MPB.”*	2 (3%)	4 (7%)
	No significant impact	*AA: “I'm fine, we need to feel grateful… your hair is not a priority it's just something it don't take the control of your life you still in charge of what or what not want to affect you.”*	8 (11%)	3 (5%)

### 3.5 Role of sexual orientation

Fifteen percent of men with AA identified as gay, bisexual or ‘other’, compared to 20% of men with AGA. Using the imputed dataset across the whole sample, sexual minority men reported significantly higher scores on the FNAES compared to those who identified as straight (t(218.71) = −2.27, *p* = 0.024).

## 4 DISCUSSION

This study addressed the underexplored question of how men with the two most common forms of alopecia, AGA and AA, are affected by their hair loss. Mixed methods allowed us to triangulate findings and provide a richer understanding of men's experiences of alopecia in terms of their support needs and preferences, and the psychosocial impact of alopecia.

In terms of support needs and preferences, men in both the AA and AGA subsamples had mainly sought out medical and treatment information rather than psychosocial information and support. This is consistent with the literature on coping, which suggests men typically report more instrumental and emotional inhibition coping strategies in the face of stressful events than women.^25^ The type of medical support sought differed by alopecia type, with men diagnosed with AA most likely to access GPs and Dermatologists, and men with AGA most likely to utilise online hair forums. This likely reflects the normative nature of AGA as a natural ageing process rather than a health condition requiring medical attention.^26^

Overall, findings suggest that men with alopecia, and especially AA, found their treatment largely unhelpful. This was augmented by qualitative findings in which 15% of the AGA subsample volunteered accounts of unsatisfactory treatments, most commonly in terms of expense and side‐effects. Ten per cent of men with AA also volunteered accounts of unsatisfactory treatments, mainly relating to unclear prognosis and treatment options from health professionals. In both instances positive treatment experiences were less commonly volunteered. This suggests the types and sources of support and information men with alopecia are seeking out are often not meeting their needs. It also implies that participants may have held unrealistically high expectations of treatment, possibly related to masculine norms of control and instrumental goal‐oriented behaviours^25^ as well as unregulated and co‐opted online resources.^27^ Carefully managing affected men's outcome expectations appears to be an important aspect of providing information on treatment and prognosis. Future research should also explore affected men's experiences and expectations regarding hair restoration procedures that are becoming increasingly popular.

Findings regarding the psychological impact of alopecia also give insight into men's support needs. Despite only a minority of men across both subsamples having reported accessing psychosocial information or support, notably the combined data indicated that many participants' wellbeing had been impacted. In terms of the quantitative findings, although comparative statistical analysis was not possible, on average participants scored in a lower threshold of wellbeing in terms of life satisfaction, purpose and happiness, and in a higher anxiety threshold than a matched population. The qualitative findings, in which over half of men from both groups talked about negative impact on wellbeing and confidence, shed light into psychosocial experiences of participants. Men with AA often discussed their depleted confidence and wellbeing in terms of a disrupted identity from the shock of sometimes sudden hair loss, and a sense of emasculation from losing facial and body hair. Roughly a third of men with AGA talked about their depleted confidence in terms of feeling less attractive, though this was typically described in terms of an ageing appearance, in contrast to emasculation in men with AA.

Together, these qualitative and quantitative findings clearly show that many men had experienced some form of psychological impact from alopecia, which makes the finding that only a minority had sought out information or help for the psychological effect of hair loss stark in contrast. This discrepancy could reflect the dominance of traditional masculinity norms that discourage support‐seeking^28^ as well as minimal support options tailored for affected men^27^, ^29^ Coproducing appealing support and information resources with and for men who have alopecia, tailored to the gendered experiences of men, is vital to addressing the gap. As an example, [charity] have since produced men's resources, including a short film featuring four men with alopecia discussing their experiences.

Findings also illuminate the perceived social impact of alopecia on men, as well as their preferred strategies for managing this impact. In the open‐ended question, a quarter of men with AA reported impacted social functioning, and the same number described using concealment strategies to manage this, mainly by wearing hats or growing hair (when possible) to cover patches. Unlike women with AA, no men reported using wigs. At the intersection between psychological and social impact, participants in both subsamples reported that although concealment strategies had aided their social functioning, these strategies did not alleviate their appearance anxieties, which in turn may impinge their ability to fully engage in social activities. This builds on research on wig use as the predominant concealment strategy in women with alopecia, in which the authors found that concealment may simultaneously reduce fear of negative evaluation while maintaining anxiety about accidently revealing their alopecia.^6^ Providing men with a range of strategies for telling others about their alopecia, in ways that promote their control over disclosure, may empower men who feel ready to take this approach. Doing so should reduce such anxiety around ‘exposure’.^30^

Consistent with Jankowski and colleagues (2021),^27^ results also indicate that for men with AGA, shaving one's head often helps to expedite adjustment to hair loss and reduces anxiety, while this was reported less by men with AA. It may be that growing or grooming facial hair and/or body hair can offer alternative strategies for managing appearance and a sense of masculinity in men with AGA, which is less feasible for men with AA, especially for those with alopecia universalis.

Findings also suggest that men with AA and AGA who identify as gay, bisexual or another sexual minority are more vulnerable to anxiety about their appearance from hair loss. This should be borne in mind when creating and offering psychosocial support content for men with alopecia; for example, it may be helpful to acknowledge the centrality of appearance ideals in gay culture.^18^ Further research into the intersection of sexuality, masculinity and visible differences in men is warranted.

### 4.1 Limitations

In terms of limitations, the high percentage of the sample who had sought out information or treatment for their alopecia may constrain its generalisability to all men with alopecia, given low overall medical help‐seeking rates in men.^17^ This may reflect the recruitment strategies employed in the study, favouring men who had engaged with support organisations or online forums. Further research should seek to understand the experiences of men with alopecia less engaged in support, for example, via recruitment strategies using community outreach methods. It should also be acknowledged that the qualitative question on participants' experiences involved volunteering information that came to mind as particularly noteworthy. We cannot conclude, therefore, that individuals who did not mention a particular theme had no significant experience related to that theme.

## 5 CONCLUSIONS

This study offers new insights into the psychosocial impact of the most common forms of alopecia in men. By integrating quantitative and qualitative findings, the results point to affected men experiencing disrupted psychosocial processes borne from cosmetic changes and self‐consciousness, as well as lower wellbeing than unaffected men, while simultaneously engaging in minimal help‐seeking for such concerns. This indicates an unmet need requiring redress from researchers and health professionals. In addressing men's psychosocial needs, this study's findings also suggest sexual minority status as a psychosocial risk factor, which should be recognised and accounted for.

## FUNDING INFORMATION

This work was supported by a small grant from the Vocational Training Charitable Trust Foundation.

## CONFLICT OF INTEREST STATEMENT

None to declare.

## AUTHOR CONTRIBUTIONS

**Fabio Zucchelli**: Conceptualization (equal); formal analysis (equal); funding acquisition (supporting); investigation (equal); methodology (equal); project administration (equal); writing – original draft (equal); writing – review & editing (lead). **Abbi Mathews**: Visualization (lead); writing – original draft (equal); writing – review & editing (supporting). **Nick Sharratt**: Conceptualization (equal); formal analysis (equal); funding acquisition (supporting); investigation (equal); methodology (equal); project administration (equal); writing – review & editing (supporting). **Kerry Montgomery**: Funding acquisition (lead); investigation (supporting); writing – review & editing (supporting). **Jen Chambers**: Funding acquisition (lead); investigation (supporting); project administration (supporting); writing – review & editing (supporting).

## ETHICS STATEMENT

Ethical approval was gained from the University of the West of England Health and Applied Sciences Faculty Ethics Committee (HAS.20.07.201).

## PATIENT CONSENT

Not applicable.

## Supporting information

Table S1

## Data Availability

The data underlying this article will be shared on reasonable request to the corresponding author. The data are not publicly available due to privacy or ethical restrictions.

## References

[1] Rathnayake D , Sinclair R . Male androgenetic alopecia. Expert Opin Pharmacother. 2010;11(8):1295–1304. 10.1517/14656561003752730 20426708

[2] Harries M , Macbeth AE , Holmes S , Chiu WS , Gallardo WR , Nijher M , et al. The epidemiology of alopecia areata: a population‐based cohort study in UK primary care. Br J Dermatol. 2021;186(2):257–265. 10.1111/bjd.20628 34227101 PMC9298423

[3] Messenger AG , McKillop J , Farrant P , McDonagh AJ , Sladden M , Hughes J , et al. British Association of Dermatologists’ guidelines for the management of alopecia areata 2012. Br J Dermatol. 2012;166(5):916–926. 10.1111/j.1365-2133.2012.10955.x 22524397

[4] Kanti V , Messenger A , Dobos G , Reygagne P , Finner A , Blumeyer A , et al. Evidence‐based (S3) guideline for the treatment of androgenetic alopecia in women and in men – short version. J Eur Acad Dermatol Venereol. 2018;32(1):11–22. 10.1111/jdv.14624 29178529

[5] Fukumoto T , Fukumoto R , Magno E , Oka M , Nishigori C , Horita N . Treatments for alopecia areata: a systematic review and network meta‐analysis. Dermatol Ther. 2021;34(3):e14916. 10.1111/dth.14916 33631058

[6] Montgomery K , White C , Thompson A . A mixed methods survey of social anxiety, anxiety, depression and wig use in alopecia. BMJ Open. 2017;7(4):e015468. [cited 2021 Dec 2]. 10.1136/bmjopen-2016-015468 PMC5566602 28473521

[7] Stock NM , Sharratt ND , Treneman‐Evans G , Montgomery K , Denman R , Harcourt D , et al. My face in someone else’s hands’: a qualitative study of medical tattooing in women with hair loss. 2021. [cited 2021 Dec 2]. 10.1080/1354850620211883688 33559487

[8] Davey L , Clarke V , Jenkinson E . Living with alopecia areata: an online qualitative survey study. Br J Dermatol. 2019;180(6):1377–1389. 10.1111/bjd.17463 30501016

[9] Macbeth AE , Holmes S , Harries M , Chiu WS , Tziotzios C , de Lusignan S , et al. The associated burden of mental health conditions in alopecia areata: a population‐based study in UK primary care. Br J Dermatol. 2022;187(1):73–81. [cited 2022 Apr 13]. https://onlinelibrary.wiley.com/doi/full/10.1111/bjd.21055 35157313 10.1111/bjd.21055 PMC9542942

[10] Razum J . Cosmetic TVHJ of, 2021 undefined. Quality of life in young men with androgenetic alopecia: a mixed methods study. Wiley Online Libr; 2021. [cited 2021 Dec 8]. https://onlinelibrary.wiley.com/doi/abs/10.1111/jocd.14132 10.1111/jocd.14132 33817940

[11] Creadore A , Manjaly P , Li SJ , Tkachenko E , Zhou G , Joyce C , et al. Evaluation of stigma toward individuals with alopecia. JAMA Dermatol. 2021;157(4):392–398. 10.1001/jamadermatol.2020.5732 33688916 PMC7948115

[12] Gonul M , Cemil BC , Ayvaz HH , Cankurtaran E , Ergin C , Gurel MS . Comparison of quality of life in patients with androgenetic alopecia and alopecia areata. An Bras Dermatol. 2018;93(5):651–658. 10.1590/abd1806-4841.20186131 30156613 PMC6106669

[13] Rencz F , Gulacsi L , Péntek M , Wikonkal N , Baji P , Brodszky V . Alopecia areata and health‐related quality of life: a systematic review and meta‐analysis. Br J Dermatol. 2016;175(3):561–571. 10.1111/bjd.14497 26914830

[14] Barkauskaite R , Serapinas D . Therapeutic implications of psychological state in patients with alopecia areata: a qualitative study. Dermatol Ther. 2020;33(6):e14269. 10.1111/dth.14269 32882084

[15] Tucker P . Bald is beautiful?: the psychosocial impact of alopecia areata. J Health Psychol. 2009;14(1):142–151. 10.1177/1359105308097954 19129346

[16] Jankowski GS , Gough B , Fawkner H , Halliwell E , Diedrichs PC . Young men’s minimisation of their body dissatisfaction. Psychol Health. 2018;33(11):1343–1363. 10.1080/08870446.2018.1496251 30334461

[17] Yousaf O , Grunfeld EA , Hunter MS . A systematic review of the factors associated with delays in medical and psychological help‐seeking among men. Health Psychol Rev. 2015;9(2):264–276. 10.1080/17437199.2013.840954 26209212

[18] Alleva JM , Paraskeva N , Craddock N , Diedrichs PC . Body appreciation in British men: Correlates and variation across sexual orientation. Body Image. 2018;27:169–178. 10.1016/j.bodyim.2018.09.004 30292836

[19] Gibson A , Welsman J , Britten N . Evaluating patient and public involvement in health research: from theoretical model to practical workshop. Health Expect. 2017;20(5):826–835. 10.1111/hex.12486 28664563 PMC5600246

[20] Lundgren JD , Anderson DA , Thompson JK . Fear of negative appearance evaluation: development and evaluation of a new construct for risk factor work in the field of eating disorders. Eat Behav. 2004;5(1):75–84. 10.1016/s1471-0153(03)00055-2 15000956

[21] Benson T , Sladen J , Liles A , Potts HWW . Personal Wellbeing Score (PWS)—a short version of ONS4: development and validation in social prescribing. BMJ Open Qual. 2019;8(2):e000394. 10.1136/bmjoq-2018-000394 PMC6542444 31206049

[22] Zucchelli F , Donnelly O , Rush E , White P , Gwyther H , Williamson H , et al. An acceptance and commitment therapy prototype mobile program for individuals with a visible difference: mixed methods feasibility study. JMIR Form Res. 2022;6(1):e33449. 10.2196/33449 35060908 PMC8817209

[23] Newman DA . Missing data: five practical guidelines. Organ Res Methods. 2014;17(4):372–411. 10.1177/1094428114548590

[24] Miles MB , Huberman AM . Qualitative data analysis: an expanded sourcebook. 1st ed. New York: SAGE Publications; 1994.

[25] Matud MP . Gender differences in stress and coping styles. Personal Individ Differ. 2004;37(7):1401–1415. 10.1016/j.paid.2004.01.010

[26] Jankowski GS , Frith H . Psychology’s medicalization of male baldness. J Health Psychol. 2022;27(9):2161–2180. 10.1177/13591053211024724 34154437 PMC9353973

[27] Jankowski GS , Sherwin M , Deighton‐Smith N , Bell B . Just shave it off: a thematic analysis of men’s baldness forums. Int J Mens Soc Community Health. 2021;4(1):e54–e67. 10.22374/ijmsch.v4i1.55

[28] Hunt K , Adamson J , Galdas P . Gender and help‐seeking: towards gender‐comparative studies. Palgrave Handb Gend Healthc. 2010;1:207–221.

[29] Zucchelli F , Sharratt N , Montgomery K , Chambers J . Men’s experiences of alopecia areata: a qualitative study. Health Psychol Open. 2022.

[30] Sharratt ND , Williamson H , Zucchelli F , Kiff J . Becoming known: disclosure and exposure of (in)visible difference. Stigma Health. 2020;5(4):413–424. 10.1037/sah0000212

