# Supplementary material for: The psychosocial impact of alopecia in men: A mixed‐methods survey study
Source: Skin Health Dis. 2024 Jun 29;4(5):e420. doi: 10.1002/ski2.420 (PMC11442044; doi:10.1002/ski2.420)
Supplement: Supplementary file 1 — Table S1 [file SKI2-4-e420-s001.docx]

**Supplementary Table 1.** Descriptions of themes from content analysis.

|  | **Theme** | **Theme description** |
| --- | --- | --- |
| ***Internal experiences*** | **Depleted confidence & wellbeing** | Self-consciousness, dissatisfaction and preoccupation with appearance (e.g. fixation on mirror, reassurance-seeking), anxiety, depression. |
|  | **Coming to terms** | Acceptance of condition: Age and time help / Not “dwelling” / perspective. |
|  | **Perception of diminished attractiveness** | Lost confidence in dating / romantic relationships / Sense of hair loss accelerating ageing process. |
|  | **"I don't recognise myself"** | Initial or ongoing shock of having no/less hair / Lost or changed identity. |
|  | **Non-scalp hair loss especially difficult** | Loss of body hair and eyebrows, eyelashes, beard as being more distressing/emasculating than scalp hair. |
|  | **Personal growth through hair loss** | Despite challenges, ultimately hair loss was catalyst for resilience/self-knowledge/self-acceptance. |
| ***Practical experiences*** | **Social functioning impacted** | Lost friendships / Avoided or left public spaces (e.g. gym, beach, pool etc.) and people. |
|  | **Concealment strategies** | Used hat, hood, or style remaining hair to manage anxiety / feel more confident. |
|  | **Work / education impacted** | Left or paused school/college/employment / Difficulties with employers or colleagues  / Changed career or job / Want to work from home. |
|  | **Physical difficulties from hair loss** | Cold / sweat from loss of eyebrows / dirt in eyes from no eyelashes / wearing hat in heat, burning easily. |
|  | **Breakthrough from shaving head** | Shaving head fostered confidence / acceptance / is socially acceptable/fashionable in current times. |
| **Influence of others** | **Social support** | Supportive/accepting partner/friends/family / community |
|  | **Unsolicited attention** | Staring / comments / teasing / harassment. |
|  | **Hair loss misunderstood** | Lack of support/understanding from general public, including confounding AA with AGA / well-meaning but unhelpful comments. |
|  | **Assumptions of illness** | Others assuming hair loss due to cancer treatment / other condition / fear over people assuming hair loss due to illness. |
| **Comparators** | **Hair loss hardest when young** | |
|  | **AA & AGA differences** | AGA more accepted / ‘normal’ / predictable. |
| **Treatment** | **Unsatisfactory treatment** | Passive or uninformed HPs / Exploitation of personal suffering / unpleasant experiences. |
|  | **Helpful treatment** | At least partially effective or helpful treatment |
|  | **No significant impact** | Minimal/no impact, other concerns being viewed as more important than AA/AGA. |
